# Role of intraoperative Iso-C based navigation in challenging spine trauma

**DOI:** 10.4103/0019-5413.36993

**Published:** 2007

**Authors:** Ashish Jaiswal, Ajoy P Shetty, S Rajasekaran

**Affiliations:** Department of Spine Surgery, Ganga Hospital, Coimbatore, India

**Keywords:** Computer-assisted surgery, neuronavigation, pedicle screw, spine fracture, challenging spinal trauma

## Abstract

**Background:**

Pedicle screw fixation is the most preferred method of stabilizing unstable spinal fractures. Pedicle screw placement may be difficult in presence of fractured posterior elements, deformed spine, gross instability and spinal pathology. Challenging spine-fracture fixation is defined as the presence of one or more of the following: 1) obscured topographical landmarks as in ankylosing spondylitis, 2) fractures in occipitocervical or cervicothoracic regions and 3) preexisting altered spinal alignment. We report a series of pedicle screw insertion with guidance of navigation in difficult fixation problems..

**Materials and Methods:**

Fourteen patients [hangman's fracture (n=3), odontoid fracture (n=4), C1C2 fracture (n=1) and spinal fracture with coexistent ankylosing spondylitis (n=6)] underwent posterior stabilization. Intraoperatively after surgical exposure, images were acquired by Iso-C 3D C-arm and transferred to navigation system. Instrumentation was performed with navigational assistance. Postoperatively, placements of pedicle screws were evaluated with radiographs and CT scan.

**Results:**

Sixty-seven pedicle screws (cervical, n=33; thoracic, n=6; lumbar, n=26; sacral n=2) and 15 lateral mass screws were inserted with navigation guidance. The average time of image data acquisition by Iso-C 3D C-arm and its transfer to workstation was 4 minutes (range, 2-6 minutes). Postoperative CT scan revealed ideal placement of screws in 63 pedicles (94%), grade 1 cortical breaches (<2 mm) in 3 pedicles (4.5%) and grade 2 cortical breach (2-4 mm) in one pedicle (1.5%). There were no neurovascular complications. Deep infection was encountered in one case, which settled with debridement.

**Conclusions:**

Intraoperative Iso-C 3D C-arm based navigation is a useful adjunct while stabilizing challenging spinal trauma, rendering feasibility, accuracy and safety of pedicle screw placement even in difficult situations.

Although majority of spine fractures can be treated conservatively, surgical stabilization is warranted in grossly unstable fractures and in presence of concomitant neurological deficits.^1^,^2^ Pedicle screw has emerged as the most preferred modality of posterior spinal stabilization because of its superior biomechanical characteristics, feasibility in presence of disrupted posterior elements and possibility of concomitant decompression.^3^ Safety concerns do persist, as any misplacement can injure the adjacent visceral and neurovascular structures. Despite progressive improvement in technique of pedicle screw placement, locating pedicle screw entry points and trajectory, especially in case of fractured posterior elements and post-traumatic deformity, can be difficult.^4^ The inaccuracies in pedicle screw insertion can place the adjacent neurovascular structures at risk because of variable spinal morphology in different individuals,^5^ difficult regions (occipitocervical^4^,^5^ and cervicothoracic^6^,^7^), preexisting deformity and spinal pathology like ankylosing spondylitis obscuring the anatomical landmarks.^8^,^9^

Computer-assisted surgery, after its introduction as frameless stereotaxy in neurosurgery in late 1980s, has found various applications in spine surgeries.^10^ Its superiority over conventional techniques in various spine disorders has been demonstrated,^11^,^12^ but still its application in spine fractures is restricted to few case reports.^13^–^15^ Widely prevalent preoperative CT-based navigation is limited by registration errors due to positional intersegmental changes during surgery, as CT is acquired in supine position while surgery is performed in prone position.^10^ This factor can be furthermore limiting in unstable spine fractures because of additional instability at fracture site.^13^ Intraoperative Iso-C 3D C-arm based navigation overcomes the problem of positional error as the images are acquired intraoperatively after completion of surgical exposure.^11^,^16^

The purpose of the present study is to evaluate the role of intraoperative Iso-C 3D C-arm based navigation in challenging spine trauma where otherwise pedicle screw placement would have been considered dangerous or impossible.

## MATERIALS AND METHODS

Challenging spine fracture fixation was defined as presence of one or more of the following: 1) obscured topographical landmarks as in ankylosing spondylitis, 2) fractures in occipitocervical or cervicothoracic regions and 3) preexisting altered spinal alignment. From February 2005 through January 2007, fourteen patients [Table 1] with challenging spine fracture were operated using intraoperative Iso-C 3D C-arm based navigation. Ethics committee approval and informed consent from all the patients were obtained.

**Table 1:** Demographical, clinical and instrumentation details of patients

Age/Sex	Diagnosis	Neurology	Pedicle screws	Additional screws	Levels
58 yrs/M	C1C2 #	ASIA-E	4	Nil	C1-C2
58 yrs/F	Odontoid # nonunion	ASIA-C	4	Nil	C1-C2
69 yrs/F	Odontoid # nonunion	ASIA-D	4	Nil	C1-C2
17 yrs/M	Odontoid#, atlas #	ASIA-E	1	4 LM	O-C4
40 yrs/M	Hangman's #	ASIA-E	2	Nil	C2
55 yrs/M	# Dislocation L3,4;AS	ASIA-E	8	Nil	L2-L5
35 yrs/M	Odontoid # nonunion	ASIA-E	4	Nil	C1-C2
70 yrs/M	# Dislocation L4,5;AS	ASIA-E	12	Nil	L2-S1
43 yrs/M	C2,3 subluxation; AS; kyphotic deformity	ASIA-E	2	3LM	O- C5
47 yrs/M	# Dislocation L3,4;AS	ASIA-C	8	Nil	L2-5
45 yrs/M	# subluxation C6,7; AS	ASIA-C	10	Nil	C5-T3
58 yrs/M	Chance # C3,4; AS	ASIA-B	3	4 LM	C2-C6
55 yrs/M	Hangman's #	ASIA-E	3	4LM	C1-C4
28 yrs/M	Hangman's #	ASIA-E	2	Nil	C2

C-cervical, AS-ankylosing spondylitis,

# -fracture, LM-lateral mass, O-occipital, T-thoracic, L-lumbar, S-sacral, ASIA-American Spine Injury Association Score)

The study group [Table 1] consisted of hangman's fracture (n=3), odontoid fracture nonunion (n= 4), C1C2 fracture (n=1). Six patients of ankylosing spondylitis had subluxation of C_2-3_ and C_6-7_ in one case each, chance fracture of C_3_ in one patient and fracture distraction lumbar spine L_3-4_ in two cases and L_4-5_ in one case. The mean age of patients was 48 years (range, 17-70 years), and 12 of them were male.

Preoperative workup included thorough clinical assessment, radiographs, multislice CT scans and magnetic resonance imaging. Five patients had neurological deficits (odontoid fracture nonunion – 2; coexistent ankylosing spondylitis – 3), and remaining nine were neurologically intact [Table 1].

**Figure 1:**
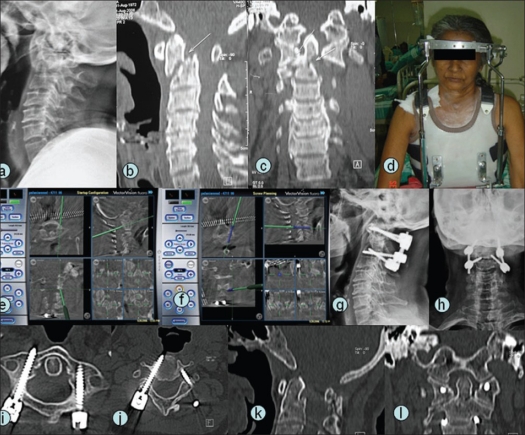
A 58-year-old female (case 2) with nonunion fracture odontoid with atlantoaxial instability (a,b,c). Preoperative halo-vest traction (d) was given. Posterior instrumented C1C2 fusion was planned. Intraoperative navigation (e,f) helped in accurate localization of entry point and in ascertaining trajectory and dimensions of pedicle screws. Postoperative radiographs (g,h) showed satisfactory alignment. Ten-month follow-up CT scan (i,j) showed good placement of C1 and C2 pedicle screw and union in satisfactory alignment (k,l).

**Figure 2:**
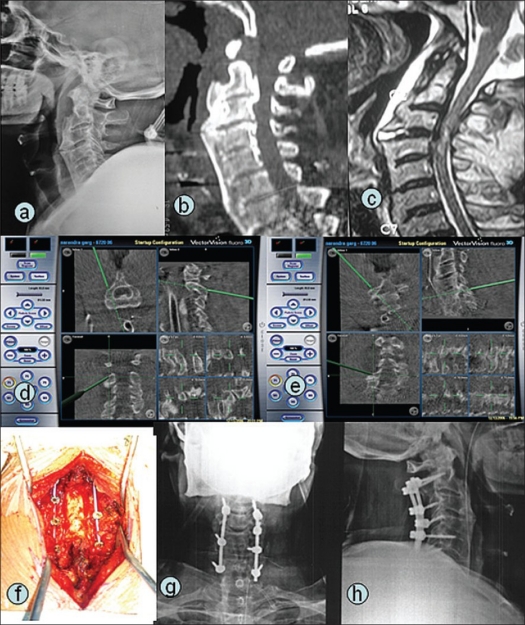
A 58-year-old male (case 12) with ankylosing spondylitis sustained chance fracture at C3-C4 (a,b,c) with ASIA grade B neurological deficit. Posterior instrumented fusion and decompression was planned. Intraoperative navigation pictures (d,e) showing multiplanar pedicle entry point and trajectory localization. Intraoperative photograph (f) and postoperative radiographs showing good placement of pedicle and lateral mass screws (g,h)

Preoperative planning was done based on radiological characteristics of fractures [Table 1]. C1-C2 fixation was scheduled for a case of C1-C2 fracture (case 1) and three cases of odontoid nonunion (cases 2, 3, 7) [Figure 1]; and in one odontoid nonunion with concomitant atlas fracture, occipitocervical fixation was planned (case 4). Osteosynthesis was provisionally planned for two of hangman's fractures (cases 5, 14); while in the third one, communition of fracture warranted segmental fixation (case 13). Long-segment fixation was scheduled for fracture fixation in all patients of ankylosing spondylitis (cases 6, 8, 9, 10, 11, 12, Figure 2).

### Surgical technique

All the patients were operated in prone position on a carbon fiber radiolucent operating tabletop under general anesthesia. Patients were positioned in such a manner that relevant anatomy was in isocenter (i.e., at midpoint of arc of rotation) of Iso-C C-arm (Siremobil Iso-C^3D^; Siemens Medical Solutions, Erlangen, Germany) in both anteroposterior and lateral views. The region to be instrumented was exposed by conventional midline posterior approach. MIRA (minimally invasive reference array) was firmly affixed to the spinous process of vertebrae next to the caudal-most vertebra to be instrumented. The computer workstation (Vector vision compact, BrainLAB-AG, Germany) with its electro-optic camera was stationed at the foot end of operating table to allow simultaneous tracking of both MIRA and calibration target attached to Iso-C. Images were acquired by Iso-C 3D C-arm with automated orbital rotation of 190°. The images were reconstructed in processor unit of Iso-C and transferred automatically to computer workstation, which obviated the need for separate manual registration. Good quality sagittal, axial and coronal reformatted images were produced on a virtual basis whenever the marker tool was brought into the field of data acquisition. Registration of spinal anatomy was verified by placing navigator tool over exposed topographical landmarks, and its correspondence with images displayed in monitor was ensured. The C-arm was then moved away from the operating field with no need for any further fluoroscopic exposures during the entire procedure.

Instruments like burr, pedicle awl and drill were calibrated to navigation system by Instrument Calibration Matrix -4, so that their position relative to patient's anatomy could be tracked during surgical procedure. Pedicle screw entry points were localized by navigator tool and developed with precalibrated 2.5 mm high-speed burr under navigational assistance. Pedicle tracts were made with navigation, ensuring proper trajectory, using precalibrated drill in cervical spine and awl in thoracolumbar spine. Additionally, integrity of pedicle tract was verified manually by tactile feedback with ball probe intermittently before incremental advancement. The length and diameter of pedicle screws were ascertained with navigational assistance. Decompressive laminectomy if needed was done after instrumentation.

Intraoperative blood loss and duration of surgery were documented. Postoperatively, periodic clinical and neurological assessment of patients was done. All the patients were mobilized with region-specific brace after the removal of drain. Postoperative CT scans were performed briefly after the surgery or in followup to assess the placement of pedicle screws. The pedicle screw placement was graded on CT as follows: Grade 0, no pedicle perforation; Grade 1, only the threads outside the pedicle (less than 2 mm); Grade 2, core screw diameter outside the pedicle (2-4 mm); Grade 3, core screw diameter outside the pedicle (more than 4 mm); and Grade 4, screw entirely outside the pedicle.^11^

## RESULTS

Intraoperatively, clear visualization of pedicles was obtained with navigation in all the cases. Sixty-seven pedicle screws (cervical, n=33; thoracic, n=6; lumbar, n=26; sacral, n=2) and 15 lateral mass screws were inserted with navigational guidance. Fifteen of the 48 cervical pedicles planned for instrumentation were considered not suitable for screw placement and were aborted owing to extremely small dimensions (n=9), fractured pedicle (n=2) or absent medullary canal (n=4). Here, fixation was achieved by placing lateral mass screws. Eleven of these aborted pedicle screws were in patients with ankylosing spondylitis. The average number of instrumented segments was 2.5 (range, 0-5). In two cases of hangman's fracture, it was possible to do osteosynthesis of fractured C2 pedicles, obviating the need for segmental fixation.

Instrumentation was done in occipitocervical spine in two patients, cervical in eight, cervicothoracic in one, lumbar in two and lumbosacral in one patient. Posterior decompressive laminectomy was done in five patients (cervical, n=4; lumbar, n=1) with post-injury neurological deficits [Table 1]. None had concomitant anterior fixation. The average time of image data acquisition by Iso-C 3D C-arm and its transfer to workstation was 4 minutes (range, 2-6 minutes). The mean duration of surgery was 130 minutes (range, 100-240 minutes), and average blood loss was 550 ml (range, 200-1100 ml).

Postoperative CT scan revealed good placement of screws in all except grade 1 cortical breaches (<2 mm) in three pedicles (4.5%) and grade 2 breach in one (1.5%). Of these pedicle breaches, three occurred in cervical spine (two grade 1 – right C1, left C5; one grade 2 – right C3) and one grade 1 breach in lumbar spine (right L2, ankylosing spondylitis). There were no neurovascular or procedure-related complications. No pedicle screws required revision for malpositioning. Deep infection was encountered in one case of ankylosing spondylitis in early postoperative period, which settled with debridement and intravenous antibiotics. Neurological recovery of at least one grade was noted in all the cases with preoperative neurological deficits.

## DISCUSSION

Pedicle screw placement is a technically demanding procedure because of complex three-dimensional morphology of vertebrae and closely located critical neurovascular structures.^5^,^17^ Various technical modifications have been suggested to improve the accuracy of pedicle screw placement.^3^,^18^,^19^ The identification of the correct anatomic landmarks, tactile feedback by probe and the use of intraoperative radiography or fluoroscopy are common methods for safe and accurate insertion of the pedicle screw.^4^ Although thoracic and lumbar spine pedicle screw freehand placement with fluoroscopic guidance has attained acceptable accuracy, its use in cervical pedicles is still considered challenging for even experienced surgeons.^4^,^5^,^18^,^19^ In the presence of ankylosing spondylitis, the topographical landmarks for pedicle entry are difficult to identify in widely ossified spine^8^,^9^; moreover, spine is usually kyphotic, thus leading to inaccuracy of pedicle screw placement even in thoracolumbar spine.

Computer-assisted surgery, since its inception as frameless stereotaxy in neurosurgery, has demonstrated improved precision in various spinal applications. Improved accuracy has been reported in pedicular screw placements with navigational assistance in stabilizing spine in various spinal disorders.^11^,^12^ Navigation provides real-time, precise, multiplanar virtual visualization of pedicle and thus gives the surgeon intraoperative guidance while placing pedicle screws. In a recent meta-analysis^20^ of 130 published studies, the median placement accuracy for the *in vivo* assisted navigation subgroup (95.2%) was higher than that of the subgroup without the use of navigation (90.3%).

Preoperative CT-based navigation is the most commonly employed system. With time, limitations of preoperatively acquired image based navigation have been realized like cumbersome surgeon-driven point-to-point registration, prerequisite of CT scan according to specific protocol and simulation error due to difference in position of scan acquisition and surgery.^10^ With availability of intraoperative Iso-C 3D C-arm based navigation, these limitations have been largely overcome.^11^,^16^ Some investigators have used intraoperative fluoroscope-based navigation,^21^ but absence of three-dimensional images limits its usefulness, particularly in complex cervical pedicles or in cervicothoracic junction.

The published literature regarding the use of navigation in spinal fractures is restricted to few case reports.^13^–^15^ Our study provides valuable information on role of Iso-C 3D C-arm based navigation in unstable spinal fractures, especially in challenging situations. Intraoperative acquisition of images after positioning and reduction of fracture solves the problem of simulation error due to change of patient position during surgery and changes due to instability at fracture site, to some extent. Still it is recommended to observe caution, not to move the spine extraordinarily while instrumentation. Use of drills can minimize the pressure needed to develop the pedicle tract, decreasing the associated movement. The rate of misplaced cervical pedicle screws in the present study, three out of 67 (4.5%), must be considered quite low, especially considering the spine fractures in patients included were relatively challenging. Despite four pedicular violations, none of these cases has clinical manifestations. This correlates with findings of other authors that majority of pedicular violations are assymtomatic.^4^ Optimal placement of pedicle screw is important for improved biomechanical purchase, besides safety concerns.

Cervicothoracic junctional region fractures pose unique problems of poor radiographic visualization and approach-related difficulty because of the anteriorly located vital structures.^6^,^7^ Posterior pedicle screw offers an easier and biomechanically apt modality to stabilize fractures in this region. Standard fluoroscopic guidance suffers in this region due to overlap of shoulders, and thence navigation becomes furthermore important. We instrumented successfully one case of fracture in cervicothoracic region in spite of coexistent ankylosing spondylitis.

Judet direct pedicular osteosynthesis is considered optimal treatment in Hangman's fracture, being a “physiological operation.”^22^ This surgery is rarely performed because of the high risk of spinal cord damage or vertebral artery tear. Direct transpedicular osteosynthesis was safely performed in two Hangman's fractures in our series, thus avoiding the instrumentation and fusion of uninjured motion segments. Arand *et al.* reported safe transpedicular screw fixation of one Hangman's fracture with preoperative CT-based spinal navigation; but they cautioned against the possibility of simulation error, especially in highly unstable fracture due to change in intervertebral anatomy after patient positioning for surgery.^13^ Taller *et al.* have described intraoperative CT-guided direct transpedicular screw fixation of 10 Hangman's fractures with excellent outcome.^23^ But it has limited applicability owing to high costs, ergonomic issues, increased radiation exposure and need of special operation suite.

Cervical spine pedicle screw placement infrequently needs to be aborted due to extremely small dimensions and obliterated medullary canal.^4^,^5^,^9^ Here, alternative modalities like lateral mass screws and wire cables may be employed. Careful selection of pedicle is important to improve accuracy of screw placement. In 15 cervical pedicles in our series, screw placement was aborted and lateral mass screw fixation was used at respective levels. In our series only one complication was noted in the form of deep infection in case of ankylosing spondylitis, but it settled with timely debridement and parenteral antibiotics. Surgery in fractures in cases with ankylosing spondylitis are known to be plagued with complications like higher blood loss, formation of epidural hematoma, surgical wound infection and pulmonary complications.^9^

In Iso-C navigation assisted surgeries, radiation to surgical team is significantly reduced as they can stay at a distance during 3D image acquisition; moreover, during actual procedure C-arm is moved out of surgical field.^11^ Although resolution in Iso-C based navigation is slightly inferior to CT-based navigation, yet it is sufficient for most of the clinical applications. Iso-C navigation does have limitations – in cases of morbid obesity, where centralization of relevant anatomy can become difficult; and in cases of severe osteopenia, where resolution is not adequate.^11^,^22^

## CONCLUSIONS

Intraoperative Iso-C 3D C-arm based navigation is a useful adjunct while stabilizing challenging spinal trauma, rendering feasibility, accuracy and safety of pedicle screw placement even in difficult situations. However, the risk of injuring the adjacent neurovascular structures cannot be completely eliminated. Iso-C based navigation is unique in its ability to acquire multiplanar three-dimensional images of intraoperative anatomy, automated registration, simplified workflow and real-time feedback during instrumentation.
